# Supplementary material for: Highly Efficient Fluorescent Detection of Vitamin B12 Based on the Inner Filter Effect of Dithiol-Functionalized Silver Nanoparticles
Source: Nanomaterials (Basel). 2023 Aug 29;13(17):2444. doi: 10.3390/nano13172444 (PMC10490474; doi:10.3390/nano13172444)
Supplement: Supplementary file 1 [file nanomaterials-13-02444-s001.zip › nanomaterials-2564613-supplementary.pdf]

nanomaterials

Supporting Information

Highly Efficient Fluorescent Detection of Vitamin B_{12} Based on the Inner Filter Effect of Dithiol-Functionalized Silver Nanoparticles

Phan Ba Khanh Chau †, Trung Hieu Vu † and Moon Il Kim *

Department of BioNano Technology, Gachon University, Seongnam, Gyeonggi 13120, Republic of Korea; khanh.chau.0767@gmail.com (P.B.K.C.); hieu.vutrung24596@gmail.com (T.H.V.)
* Correspondence: moonil@gachon.ac.kr; Tel.: +82-31-750-8563
† These authors contributed equally to this work.

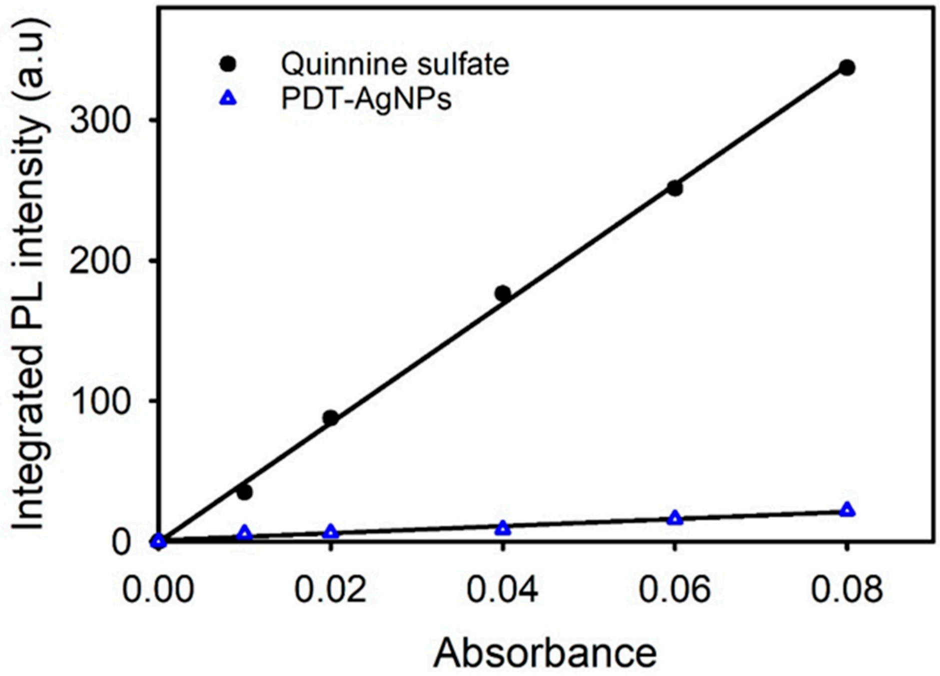

Figure S1. Measurement of quantum yield of PDT-AgNPs.

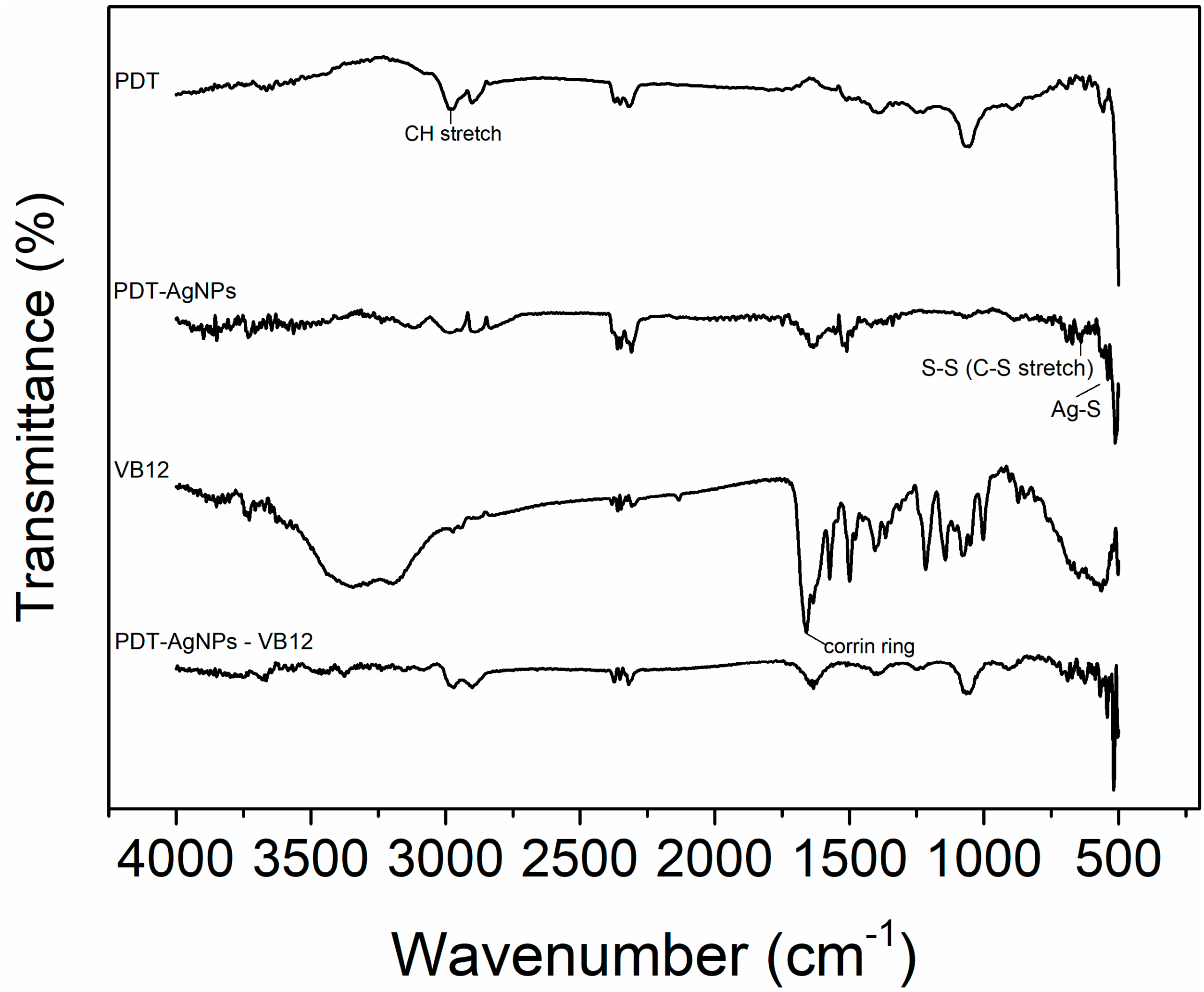

Figure S2. FT-IR spectra of PDT, PDT-AgNPs, VB_{12}, and the mixture of PDT-AgNPs with VB_{12}.

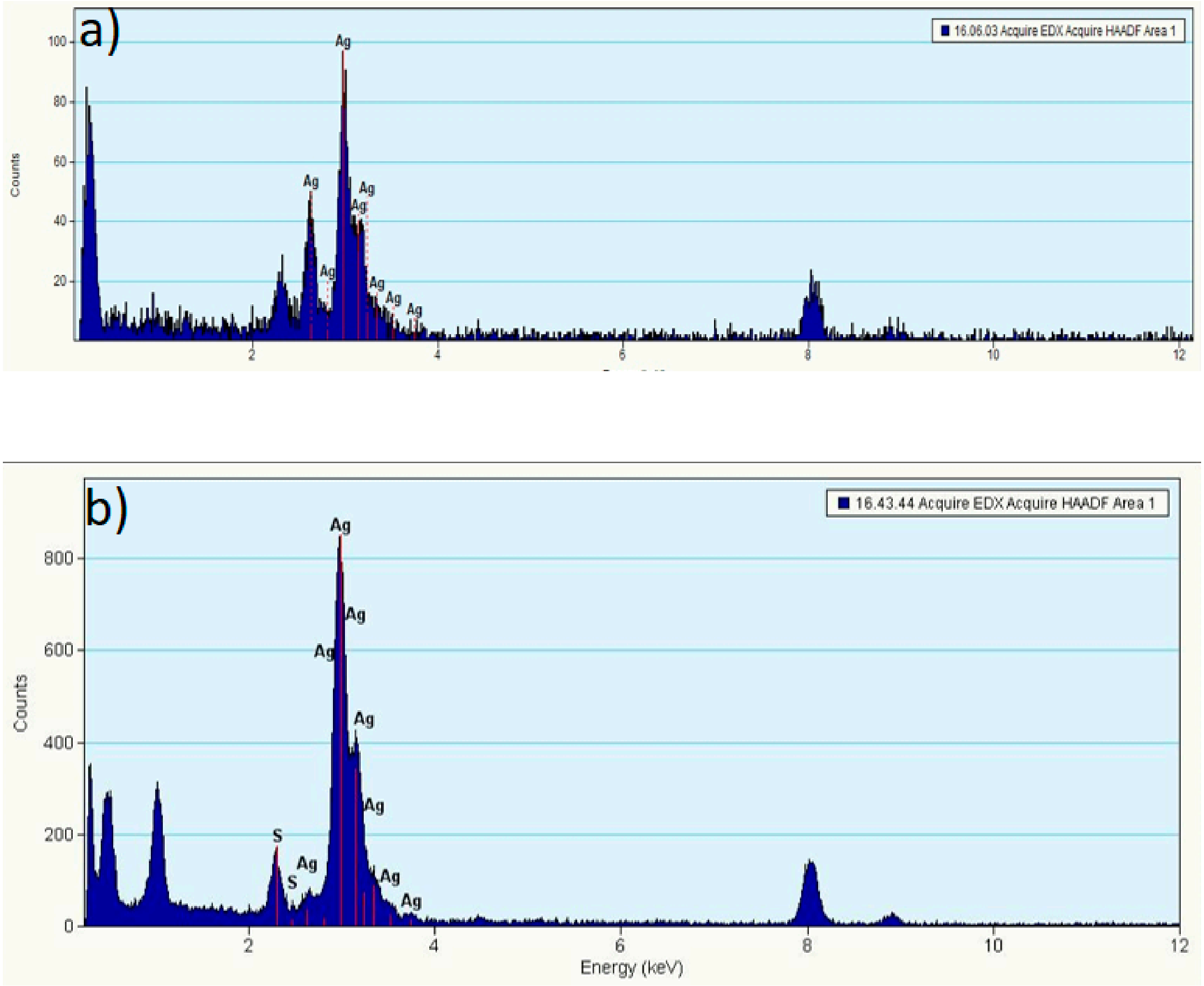

Figure S3. EDS spectra for a) bare AgNPs and b) PDT-AgNPs.

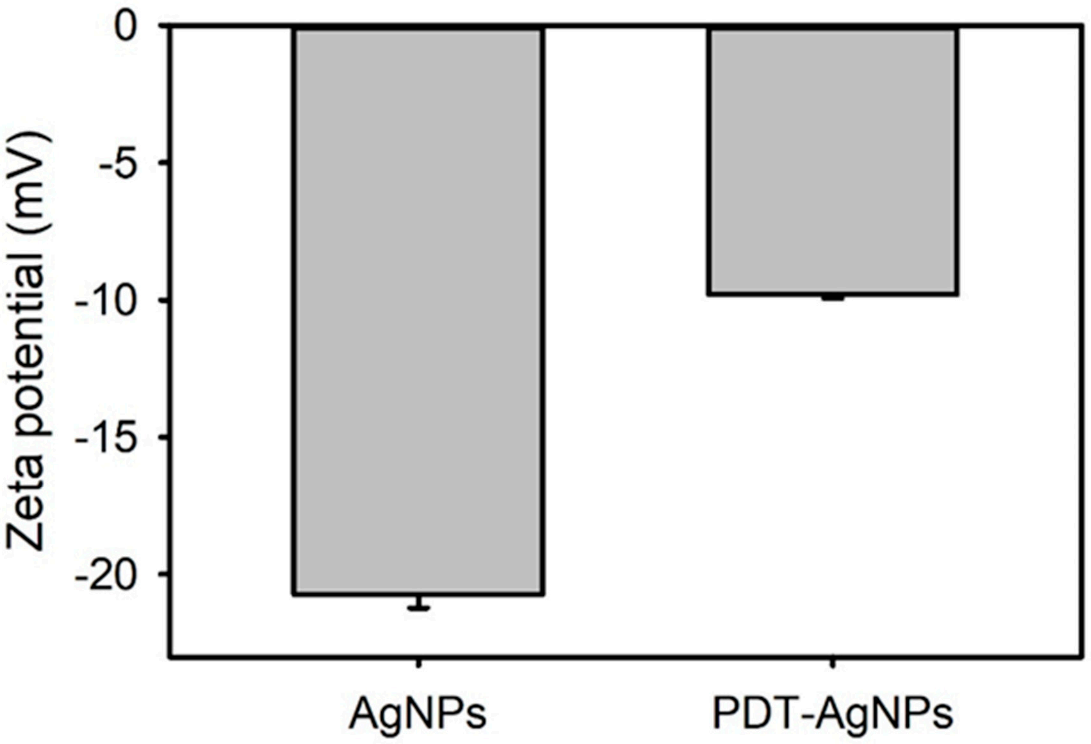

Figure S4. Surface zeta potentials of AgNPs and PDT-AgNPs (n=5).

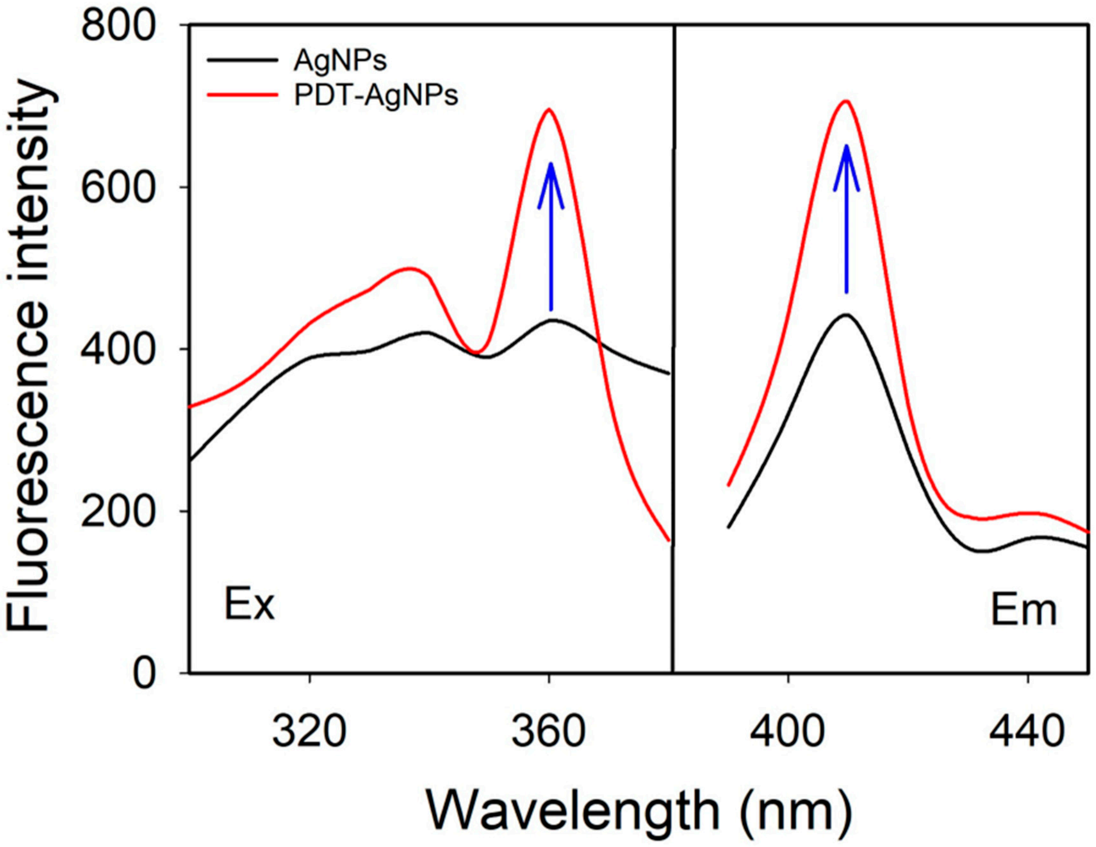

Figure S5. Excitation (left) and emission (right) scanning of bare AgNPs and PDT-AgNPs.

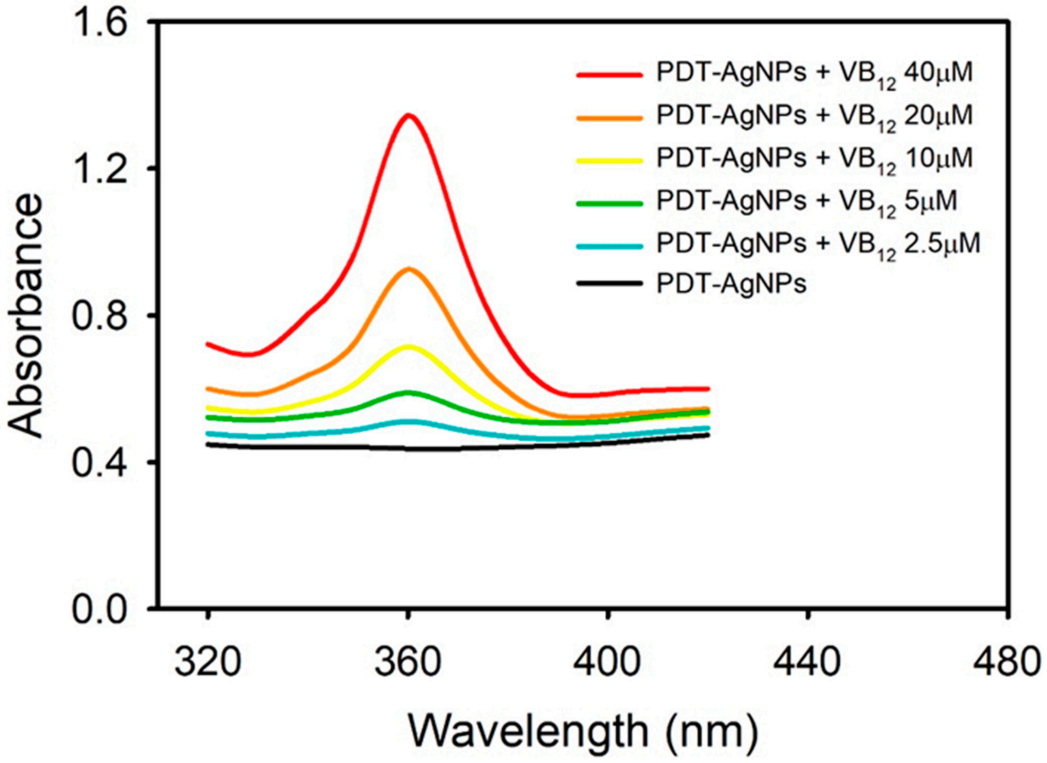

Figure S6. Excitation spectra of PDT-AgNPs in the presence of VB_{12} at diverse concentrations.

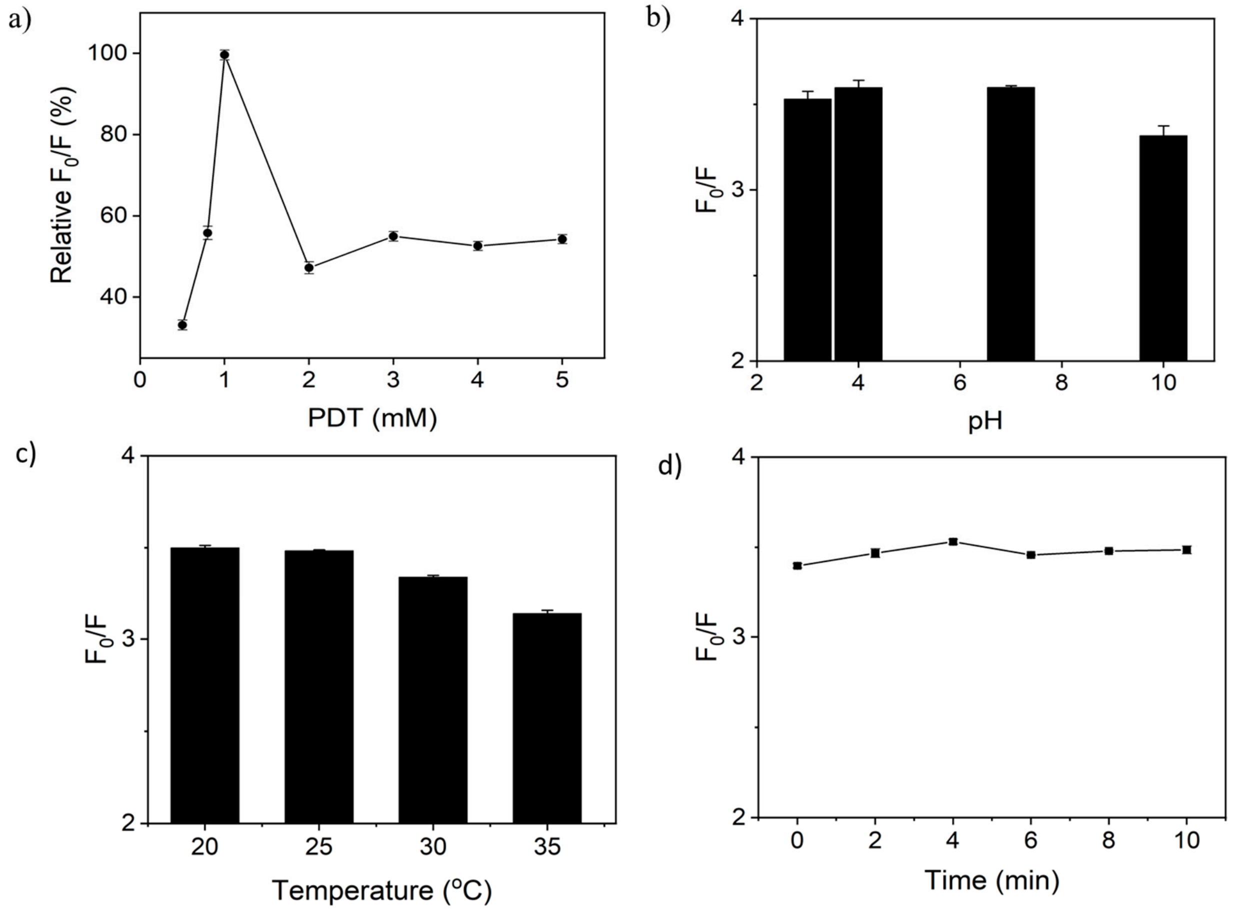

Figure S7. Effects of a) PDT concentration, b) pH, c) temperature, and d) reaction time, on fluorescence quenching efficiency of PDT-AgNPs toward VB_{12}.

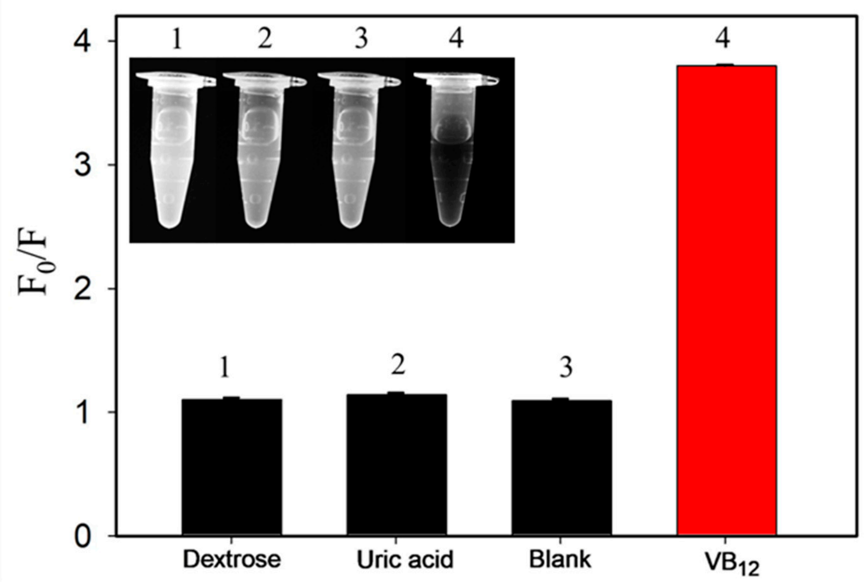

Figure S8. Additional selectivity assay toward VB_{12}.

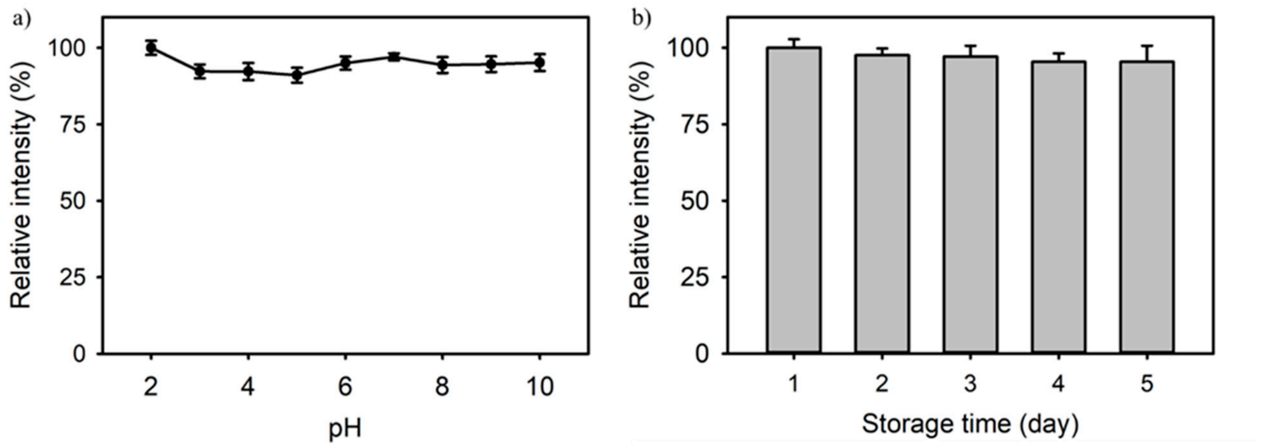

Figure S9. Stabilities of PDT-AgNPs-mediated system in ranges of a) pH and b) storage time.

Table S1. Quenching constants for the reaction of PDT-AgNPs and VB_{12} at different temperatures.

**Temperature**	**K_{sv} (L mol^{-1})**	**R^2**
298 K	$1.737x10^4$	0.994
308 K	$1.69x10^4$	0.991
318 K	$1.95x10^4$	0.993
